# Coaching programmes in undergraduate medical education: An integrative review protocol

**DOI:** 10.12688/f1000research.179726.2

**Published:** 2026-07-14

**Authors:** Lucia Weber, Jodie Freeman, Stefan Gysin, Jonas Florin, Anders Sondén, Dominik Schaer, Sissel Guttormsen

**Affiliations:** 1 Institute for Medical Education, University of Bern, Bern, Switzerland; 2 Faculty of Medicine, University of Zurich, Zurich, Switzerland; 3 Faculty of Health Sciences and Medicine, University of Lucerne, Lucerne, Switzerland; 4 Department of Clinical Science and Education, Karolinska Institutet, Stockholm, Sweden; 5 Department of Internal Medicine, University Hospital Zurich, Zurich, Switzerland

**Keywords:** Coaching, undergraduate medical education, curriculum development, medical student, review

## Abstract

**Introduction:**

Coaching programmes in undergraduate medical education are increasingly implemented to support students’ professional and personal development. Yet, the design and purpose of these programmes vary significantly across different institutions. This integrative review will systematically synthesise the literature on coaching programmes, focusing on structural characteristics, goals and educational strategies.

**Methods and analysis:**

A comprehensive literature review will be conducted using Whittemore and Knafl’s integrative review methodology. The search strategy includes the following electronic databases: MEDLINE, Embase, PsycINFO, ERIC, Education Research Complete, supplemented by a hand search. Two reviewers will independently screen all retrieved records, and study selection will be documented in a PRISMA flowchart. Data will be extracted from eligible full texts on descriptive metadata, structural characteristics, goals and educational strategies of the described coaching programmes. The quality of the included studies will be appraised using the Mixed Methods Appraisal Tool for empirical studies and the JBI Checklist for Text and Opinion for non-empirical studies. Extracted data will be analysed descriptively and thematically and integrated into a framework that can serve future programme development.

**Ethics and dissemination:**

As this review is based exclusively on secondary data, no ethical approval is required. The results of the review will be submitted to a peer-reviewed journal and presented at an academic conference.

Open Science Framework registration DOI 10.17605/OSF.IO/UTHJC

## Introduction

Undergraduate medical education places considerable cognitive, organisational and emotional demands on students. Medical students are required to manage large volumes of learning content, develop their professional identity and make early career decisions, often while navigating stress, uncertainty and continuous assessment.

Around one-third of medical students experience mental health problems such as burnout, anxiety and depression. ^1^ Poor mental health has been associated with impaired academic performance, thoughts of dropping out of medical school, reduced professionalism and empathy, medical errors, substance abuse, and suicidal ideation. ^1, 2^

In parallel, the shift towards competency-based medical education places increasing emphasis on learners’ ability to direct and regulate their own learning. ^3^ Self-regulated learning skills are essential not only for managing the immediate demands of undergraduate training, but also for developing the adaptive expertise required in the rapidly evolving healthcare environment. ^3– 5^

In the context of these challenges, coaching has emerged as an educational approach to support students’ learning, wellbeing and professional development. Coaching can be defined as a structured, learner-centred process that supports students’ individual development through reflection, goal setting and personalised guidance. ^6^ In contrast to related concepts such as advising and mentoring, coaching uniquely focuses on stimulating students’ introspection and self-learning. ^7^

Coaching is used in a variety of ways within medical education. Lovell ^8^ differentiates between three categories: coaching for wellbeing and resilience; coaching for improved non-technical skills; and coaching for technical skills. More recently, the term academic coaching has gained prominence. While skills-based coaching typically involves direct observation and feedback for improving specific clinical skills, ^8^ academic coaching usually refers to guiding students’ individual development by reviewing assessment data and personal experiences to foster reflective processes and self-regulated learning. ^5, 6^

Although coaching programmes are becoming more common in undergraduate medical education, their *structural characteristics*, *goals* and *educational strategies* vary significantly across institutions. ^9, 10^ Variation in *structural characteristics* refers to differences in how the programmes are organised, such as their format (individual versus group-based), duration, session frequency and the profile of coaches involved. Differences in *goals* relate to the intended developmental focus of the programmes, which may range from academic performance to wellbeing, professional identity formation or development of learning skills. ^10^ Variation in *educational strategies* reflects the diverse approaches used to support students’ development, including the coaching activities and any theoretical frameworks the coaching is based on.

This heterogeneity can make it difficult for educators to understand what constitutes a coaching programme, how such programmes are designed and which components are essential. However, there is limited synthesised evidence that maps this variation in a systematic way. Although prior studies provide a basic overview of coaching in medical education, ^8– 10^ there is neither a recent nor a comprehensive review on coaching programmes in undergraduate medical education.

### Objective

This review aims to synthesise literature on coaching programmes in undergraduate medical education, analysing programme structures, goals, and educational strategies to provide a foundation for future programme development.

### Research questions

The following research questions (RQ) will guide this review:

• RQ1: What are the structural characteristics of coaching programmes in undergraduate medical education?
• RQ2: What goals do these coaching programmes aim to achieve?
• RQ3: What educational strategies are used to operationalise these goals?

## Methods and analysis

We will conduct an Integrative Review, following Whittemore and Knafl’s ^11^ five-step framework: 1) problem identification, 2) literature search, 3) data evaluation, 4) data analysis, and 5) presentation. An integrative approach was chosen as it is suitable for general reviews and initial synthesis of emerging topics, allowing inclusion of various research types and enabling integration of evidence into new conceptual frameworks. ^12^

For the purpose of this review, we define a coaching programme as a structured, goal-oriented process, in which a coach meets regularly with medical students to guide their individual development and promote the students’ self-reflection. Our definition is adapted from the description of academic coaching. ^5, 6^ However, our definition intends to apply a broad scope, including not only coaching programmes that focus on reviewing assessment data to foster self-regulated learning but also other types of coaching, such as coaching for wellbeing.

### Eligibility criteria

Table 1 shows the inclusion and exclusion criteria for our review.

**Table 1.** Inclusion and exclusion criteria.

Criterion	Inclusion	Exclusion
**Population (Target Group of Coaching)**	- Undergraduate medical students	- Postgraduate medical students; residents; physicians - Students from other disciplines such as nursing, veterinary medicine, biomedicine - Medical faculty - Patients
**Setting**	- Medical schools or universities with a medical curriculum - worldwide	- Commercial coaching
**Intervention**	- Coaching programmes that meet our definition of coaching - The terms ‘coaching’ or ‘coach’ must be in the title and/or description of the program - Programmes that also include other types of support such as mentoring or advising will also be included, if the main focus of the programme is on coaching	- Mentoring, advising, training, tutoring, supervision, or interventions not explicitly described as coaching - Coaching that solely focuses on improving specific clinical skills
**Type of Literature**	Peer-reviewed academic articles	- Grey literature - Books and book chapters
**Study Design**	- Empirical (quantitative, qualitative, mixed methods) - Non-empirical (e.g., descriptive programme reports)	- Reviews (relevant reviews will not be included for data extraction, but will be used for handsearching reference lists)
**Focus/Content of the Article**	- Article should include a description of an existing, planned or theoretical coaching programme for medical students - should include structural characteristics, goals and/or educational strategies of the programme	- No description of structural characteristics, goals or content of a coaching programme
**Time Period**	No time restriction	
**Language**	English and German	Other Languages
**Availability**	Full text available	Full text unavailable

### Data sources

An electronic database search was conducted as part of this integrative review. The following databases were searched to ensure broad coverage of relevant literature: Medline, Embase, PsycINFO, and ERIC (all accessed via Ovid), as well as Education Research Complete (accessed via EBSCOhost). We will further perform a hand search of reference lists of eligible studies and relevant reviews to ensure completeness.

### Search strategy

A university librarian assisted in developing and refining the search strategy. The searches combined free-text keywords with controlled vocabulary terms specific to each database. An example of the complete search string used in Ovid MEDLINE is presented in Table 2. Full search strings for all databases are provided in the supplementary materials. The electronic database search was conducted on 25 August 2025. Table 3 shows the results of our literature search.

**Table 2.** Search string used in Ovid MEDLINE.

Line	Search terms
1	coach*.mp.
2	schools, medical/
3	education, medical, undergraduate/
4	((student* or school* or undergraduat*) adj5 medic*).mp.
5	2 or 3 or 4
6	1 and 5
7	limit 6 to (english or german)

**Table 3.** Search results.

Database	Number of articles found
Medline (Ovid)	626
Embase (Ovid)	1031
PsycINFO (Ovid)	247
ERIC (Ovid)	90
Education Research Complete (EBSCOhost)	162

### Selection process

All retrieved records were imported into the screening software *Rayyan* ^13^ and deduplicated, resulting in 1,400 unique records. The screening process consists of two stages: an initial title and abstract screening followed by full-text screening. Prior to formal screening, a pilot test with a random sample of 20 titles and abstracts was used to refine the eligibility criteria. Both reviewers will screen all records independently using these criteria. Any disagreements will be resolved through discussion, with a third reviewer available to assist in cases of uncertainty. Inter-rater reliability will not be calculated, as all records will be assessed by at least two reviewers and discrepancies will be resolved through consensus rather than quantified statistically. A reflexive diary will be maintained to record key decisions, reflections, and potential biases. Given the conceptual overlap between coaching and other interventions such as mentoring, the reviewers will apply the working definition of a coaching programme consistently during screening. We will only include articles describing programmes that clearly describe coaching, which involves fostering students’ self-reflection to help them achieve their goals. Although mentoring programmes can incorporate coaching methodology, mentoring focuses more on an experienced individual in the students’ field of interest providing expert insight to support their development. ^7^ Programmes described as mentoring, as well as programmes in which coaching is mentioned as an element of mentoring or other types of student support, will be excluded. Ambiguous or borderline cases will be discussed between reviewers to reach a shared interpretation. Any necessary refinements in our working definition and eligibility criteria will be documented in the reflexive diary. The study selection process will be reported using a PRISMA flowchart. ^14^

### Data extraction

Once the eligible full texts have been selected, relevant data will be extracted using a data extraction form. Data will be extracted on the following components:

• **Descriptive metadata**: Author, year of publication, journal, publication type, study design, country, institution, coaching program title
• **Structural characteristics of the described coaching programme (referring to RQ1)**: Target group, coaching format, number of students per coach, group size, group composition, group continuity, coach profile, overall duration, voluntary/obligatory, mode of delivery, frequency of sessions, duration of sessions
• **Goals of the described coaching programme (referring to RQ2):** intended developmental focus of the coaching programme
• **Educational strategies of the described coaching programme (referring to RQ3)**: Educational frameworks referenced, session activities and content, activities outside of coaching sessions

The data extraction form will be piloted by two reviewers on a sample of studies and refined as needed. One reviewer will extract data for all included studies, and a second reviewer will cross-check a subset of at least 10% in the first phase of the extraction to ensure accuracy and consistency.

### Quality appraisal

The methodological quality of all eligible empirical studies will be appraised using the Mixed Methods Appraisal Tool, ^15^ which allows for the assessment of qualitative, quantitative, and mixed-methods studies within a single framework. For non-empirical studies, quality appraisal will be based on the Joanna Briggs Institute Checklist for Text and Opinion. ^16^ Two reviewers will independently appraise the quality of all included studies. Discrepancies will be resolved through discussion, including a third reviewer available, if needed. Each study will receive a descriptive rating for each of the corresponding methodological criteria, indicating whether the criterion is met, not met or unclear. These quality ratings will be reported in a summary table to contextualise the strength and transparency of the included evidence. However, no studies will be excluded based on quality, consistent with integrative review methodology. ^11^ Studies with lower quality ratings will be marked in the extraction sheet and interpreted cautiously during synthesis.

### Data analysis and synthesis

Extracted data will be analysed using descriptive analysis to summarise and compare structural characteristics across studies. Qualitative data on programme goals and educational strategies will be analysed using thematic analysis. ^17^ A combined deductive-inductive coding approach will be used, with an initial deductive coding framework informed by a review of relevant literature and discussions within the research team. This framework will be iteratively refined and expanded with inductive codes as data are analysed. All extracted data will be coded by one researcher and emerging themes will be discussed within the research team until consensus is reached. Findings from both strands will be compared and synthesised into a framework, illustrating how coaching programmes can be designed to address various student needs and developmental goals.

### Reflexivity

The reviewers conducting the study selection and quality appraisal all have academic backgrounds in psychology (LW, JFr, and SGu) and bring complementary expertise in medical education and health-science research, including a junior researcher in medical education (LW), an experienced qualitative health researcher (JF), and a professor of medical education (SGu). The broader research team also includes physicians who are actively involved in undergraduate medical education and contribute complementary perspectives throughout the review process (SGy, JFl, DS). This review protocol originated from a broader project aiming to develop a coaching program for medical students. Through this work, several members of the broader research team had previously discussed the conceptual definitions of coaching, which informed our understanding of coaching and therefore the eligibility criteria to be applied in the review process.

### Dissemination

The findings of this integrative review will be shared through publication in a peer-reviewed journal and/or presentations at a relevant academic conference.

### Study status

The electronic database search has been completed and screening is ongoing at the time of submission. Any important protocol amendments will be documented reported in the final review manuscript.

## Ethical considerations

As this review is based exclusively on secondary data, no ethical approval is required.

## Data Availability

No data is associated with this article. Open Science Framework: Coaching programmes in undergraduate medical education: An integrative review, https://doi.org/10.17605/OSF.IO/AWDPY. CC-By Attribution 4.0 International. ^18^ Open Science Framework: PRISMA-P checklist for ‘Coaching programmes in undergraduate medical education: An integrative review’, https://doi.org/10.17605/OSF.IO/AWDPY. CC-By Attribution 4.0 International. ^18^
