# Supplementary material for: Coaching programmes in undergraduate medical education: An integrative review protocol
Source: F1000Res. 2026 Jul 14;15:663. Originally published 2026 May 4. [Version 2] doi: 10.12688/f1000research.179726.2 (PMC13554791; doi:10.12688/f1000research.179726.2)
Supplement: Peer review history

# Reviewer report: Reviewer response for version 2

**DOI:** 10.5256/f1000research.205941.r501261

**Published:** 2026-07-25

**Authors:** Marcelo Garcia Dieguez

**Affiliations:** 1 Universidad Nacional del Sur, Bahía Blanca, Argentina

Thank you for the detailed response and revisions. The dual-review screening process and consensus procedure, was specified, also  expanded the description of code development and thematic analysis, providing clearer criteria for distinguishing coaching from mentoring during study selection.

 These revisions should allow readers to have more methological clarity. I have no further substantive comments. I consider my previous concerns satisfactorily addressed and support approval of the revised article.

Is the study design appropriate for the research question?

Yes

Is the rationale for, and objectives of, the study clearly described?

Yes

Are sufficient details of the methods provided to allow replication by others?

Partly

Are the datasets clearly presented in a useable and accessible format?

Not applicable

Reviewer Expertise:

Competency-based curriculum design EPAs for undergraduate and residency, burnout/vitality in faculty, EPAs for undergraduate and residency, OSCE/ECOE assessment, Script Concordance Test, psychometrics (cut-off methods), postgraduate medical education and residency training .

I confirm that I have read this submission and believe that I have an appropriate level of expertise to confirm that it is of an acceptable scientific standard.

# Reviewer report: Reviewer response for version 2

**DOI:** 10.5256/f1000research.205941.r501260

**Published:** 2026-07-22

**Authors:** Krystal Hunter

**Affiliations:** 1 Cooper University Hospital, Camden, New Jersey, USA

In my review of version 1 of the paper, I stated the following:

 Strengths of work:

1. Great background information that provided details as to why this subject matter was and important topic. 
2. Detailed summary of the inclusion and exclusion that will be used to determine peer-reviewed papers  that would be eligible to be part of the review. 

Proposed Edits:

 While there were a lot of details that made it very straight forward for review; **one thing that was missing was the background of those who will be reviewing the papers for quality and review inclusion.  This should be added to the methods section.**

 The author did respond to me saying " We revised the protocol and added a reflexivity part, including the backgrounds of the researchers who are involved in selecting and appraising the quality of eligible articles." In reviewing the paper, the author did add a reflexivity section that detailed the credentials of reviewers.

Is the study design appropriate for the research question?

Yes

Is the rationale for, and objectives of, the study clearly described?

Yes

Are sufficient details of the methods provided to allow replication by others?

Partly

Are the datasets clearly presented in a useable and accessible format?

Not applicable

Reviewer Expertise:

Biostatistics, Survey studies

I confirm that I have read this submission and believe that I have an appropriate level of expertise to confirm that it is of an acceptable scientific standard.

# Reviewer report: Reviewer response for version 1

**DOI:** 10.5256/f1000research.198269.r484767

**Published:** 2026-06-16

**Authors:** Krystal Hunter

**Affiliations:** 1 Cooper University Hospital, Camden, New Jersey, USA

According to PRISMA, a systematic review protocol describes the rationale, hypothesis, and planned methods of the review. 

 The protocol does all the following:

 1. Describes the rationale:  The authors stated the following:

1. Given the rigors of being an undergraduate student, one third of them experience mental health problems (burnout, anxiety and depression).  This can lead to impaired academic performance, thoughts of dropping out of school, potential medial errors and other issues.
2. Coaching has emerged as an educational approach to support students’ learning, wellbeing and professional development. Coaching can be defined as a structured, learner-centred process that supports students’ individual development through reflection, goal setting and personalised guidance.

2.  Hypothesis/ Study Objectives:

 a. This review aims to synthesize literature on coaching programmes in undergraduate medical education, analysing programme structures, goals, and educational strategies to provide a foundation for future programme development.

 b. Research questions

 1. RQ1:What are the structural characteristics of coaching programmes in undergraduate medical education?

 2. RQ2:What goals do these coaching programmes aim to achieve?

 3. RQ3:What educational strategies are used to operationalise these goals

 3.  Planned Methods

 1. Integrative Review using he Whittmore and Knafl’s five-step framework

 2. Electronic database search

 3. Provided detail summary of the data extraction process where there will be two primary reviewers and one tie breaker review.

 4. Quality appraisal

                a. Will use Mixed Models Appraisal Tool

                b. Will also use Joanna Briggs Institute Checklist for Text and  Opinion

 5. Also provided a summary of the data analysis and synthesis and planned dissemination. 

 In summary, the authors provided sufficient information for a PRISMA compliant protocol.  It should be noted that further details are expected to be provided in the final report. 

 Strengths of work:

1. Great background information that provided details as to why this subject matter was and important topic. 
2. Detailed summary of the inclusion and exclusion that will be used to determine peer-reviewed papers  that would be eligible to be part of the review. 

Proposed Edits:

 While there were a lot of details that made it very straight forward for review; one thing that was missing was the background of those who will be reviewing the papers for quality and review inclusion.  I this should be added to the methods section

Is the study design appropriate for the research question?

Yes

Is the rationale for, and objectives of, the study clearly described?

Yes

Are sufficient details of the methods provided to allow replication by others?

Partly

Are the datasets clearly presented in a useable and accessible format?

Not applicable

Reviewer Expertise:

NA

I confirm that I have read this submission and believe that I have an appropriate level of expertise to confirm that it is of an acceptable scientific standard, however I have significant reservations, as outlined above.

# Response

**Published:** 2026-07-08

**Authors:** Lucia Weber

**Affiliations:** University of Berne Institute for Medical Education, Bern, Canton of Bern, Switzerland

Thank you for your feedback. We revised the protocol and added a reflexivity part, including the backgrounds of the researchers who are involved in selecting and appraising the quality of eligible articles.

# Reviewer report: Reviewer response for version 1

**DOI:** 10.5256/f1000research.198269.r487628

**Published:** 2026-06-12

**Authors:** Marcelo Garcia Dieguez

**Affiliations:** 1 Universidad Nacional del Sur, Bahía Blanca, Argentina

**Summary of the article : **The article  present a protocol for an integrative review of coaching programmes in undergraduate medical education. The protocol follows Whittemore and Knafl's five-step framework and aims to synthesise literature on structural characteristics, goals, and educational strategies of such programmes. The review addresses three focused research questions . The search has already been conducted across five electronic databases (MEDLINE, Embase, PsycINFO, ERIC, Education Research Complete), yielding 2,156 total records and 1,400 unique records after deduplication Quality appraisal will use the Mixed Methods Appraisal Tool (MMAT) for empirical studies and the JBI Checklist for Text and Opinion for non-empirical work. Synthesis will combine descriptive and thematic analysis.

**Cooments:** Genewrally method are well-described, and  the search string for MEDLINE ias well); The two-stage screening process (title/abstract then full-text) is described. and a pilot test of 20 records is mentioned

 The  data extraction domains are also enumerated in detail; quality appraisal tolls are mentiooed and are appropriately selected.

 Some atention is needed to improve the paper.The protocol describes that two reviewers will screen independently and the process for disagreements resolution is also describer, but  no mention is made of calculating or reporting inter-rater reliability.  The protocol states that "10–20%" of studies will be cross-checked by a second reviewer. Maybe this range is broad and tshould be s specify the exact percentage. About the thematic analysis, the authors mention some autors to follow as guidance for the process, but do not specify whether they will use inductive or deductive coding, or how themes will be generated and agreed upon between reviewers. Even brief clarification would strengthen replicability. Finally the authors acknowledge that distinguishing coaching from mentoring is challenging and describe a reflexive diary to document decisions. It would strengthen the protocol to briefly describe any decision rules that will guide this distinction in ambiguous cases. Short definitions around similarities and differences in the context of the review could also facilitate recplicability.

Is the study design appropriate for the research question?

Yes

Is the rationale for, and objectives of, the study clearly described?

Yes

Are sufficient details of the methods provided to allow replication by others?

Partly

Are the datasets clearly presented in a useable and accessible format?

Not applicable

Reviewer Expertise:

competency-based curriculum design EPAs for undergraduate and residency, burnout/vitality in faculty, EPAs for undergraduate and residency, OSCE/ECOE assessment, Script Concordance Test, psychometrics (cut-off methods), postgradute medical education and residency trainin

I confirm that I have read this submission and believe that I have an appropriate level of expertise to confirm that it is of an acceptable scientific standard, however I have significant reservations, as outlined above.

# Response

**Published:** 2026-07-08

**Authors:** Lucia Weber

**Affiliations:** University of Berne Institute for Medical Education, Bern, Canton of Bern, Switzerland

Thank you for your suggestions. In response, we have made several revisions:

 a) We have added a clarification stating that inter-rater reliability will not be calculated during the study selection process. All records will be screened by two reviewers, and discrepancies will be resolved through consenus discussions. (Selection process)

 b) We specified the proportion of articles for which data extraction will be cross-checked by a second review (10%). (Data extraction)

 c) We extended the description of our thematic analysis approach, including how codes will be generated and refined. (Data analysis and synthesis)

 d) We provided more detail on how coaching will be conceptually differentiated from mentoring and how eligibility decisions will be made. (Selection process)
